# The AD Knowledge Portal: A Repository for Multi‐Omic Data on Alzheimer's Disease and Aging

**DOI:** 10.1002/cphg.105

**Published:** 2020-10-21

**Authors:** Anna K. Greenwood, Kelsey S. Montgomery, Nicole Kauer, Kara H. Woo, Zoe J. Leanza, William L. Poehlman, Jake Gockley, Solveig K. Sieberts, Ljubomir Bradic, Benjamin A. Logsdon, Mette A. Peters, Larsson Omberg, Lara M. Mangravite

**Affiliations:** ^1^ Sage Bionetworks Seattle Washington

**Keywords:** Alzheimer's disease, database, genomics, open data, transcriptomics

## Abstract

The AD Knowledge Portal (adknowledgeportal.org) is a public data repository that shares data and other resources generated by multiple collaborative research programs focused on aging, dementia, and Alzheimer's disease (AD). In this article, we highlight how to use the Portal to discover and download genomic variant and transcriptomic data from the same individuals. First, we show how to use the web interface to browse and search for data of interest using relevant file annotations. We demonstrate how to learn more about the context surrounding the data, including diagnostic criteria and methodological details about sample preparation and data analysis. We present two primary ways to download data—using a web interface, and using a programmatic method that provides access using the command line. Finally, we show how to merge separate sources of metadata into a comprehensive file that contains factors and covariates necessary in downstream analyses. © 2020 The Authors.

**Basic Protocol 1**: Find and download files associated with a selected study

**Basic Protocol 2**: Download files in bulk using the command line client

**Basic Protocol 3**: Working with file annotations and metadata

## INTRODUCTION

The AD Knowledge Portal (adknowledgeportal.org) is an open access database that disseminates data, tools, and analytical results generated by several National Institute on Aging (NIA)−funded programs focused on aging, dementia, and Alzheimer's disease (AD). These large‐scale collaborative research projects, comprising more than 250 investigators, have a primary focus on discovery of targets and biomarkers for AD. The investigators generate, analyze, and evaluate multi‐scale, high‐dimensional molecular profiling data from human samples and experimental model systems. Through characterization of the molecular complexity of disease in humans and identification of appropriate experimental models for preclinical evaluation, these programs address several of the challenges that currently limit successful discovery of AD targets and biomarkers. These programs embrace open science principles and require that data and resources be released on a 6‐month cycle. This facilitates pre‐publication review of research findings to support the rapid identification of discoveries that are robustly replicated and optimally poised for translation.

The AD Knowledge Portal is designed to support transparent evaluation and broad use of resources generated within the NIA's translational research portfolio. The Portal was initially established as part of the Accelerating Medicines Partnership in Alzheimer's Disease (AMP‐AD) program (https://www.nia.nih.gov/research/amp‐ad; Hodes & Buckholtz, 2016), which was initiated in 2014 between the National Institutes of Health (NIH) and several nonprofit organizations and pharmaceutical companies. Together, this public‐private partnership has funded an open, pre‐competitive discovery effort with the goal of identifying emerging target development opportunities. The Portal has since grown to support four additional research programs focused on a broad set of questions related to AD and aging (https://adknowledgeportal.synapse.org/Explore/Programs): (1) the Molecular Mechanisms of the Vascular Etiology of AD (M^2^OVE‐AD) program; (2) the Resilience‐AD program; (3) the Psych‐AD program; and (4) the Translational Center for Model Development and Evaluation for Late Onset AD (MODEL‐AD) program.

Distribution of data through the Portal is modeled after the same open science principles established within the AMP‐AD program: all data, methods, and results generated within the network are distributed under FAIR (Findable, Accessible, Interoperable and Reusable; Wilkinson et al., 2016) principles according to the NIH Genomic Data Sharing policy (https://osp.od.nih.gov/scientific‐sharing/genomic‐data‐sharing/). In addition to resources developed within individual projects, the network also releases results from robust meta‐analysis across data sets, providing a set of high‐confidence research results that can be broadly used to support discovery efforts across the field. As of August 2020, the Portal contained more than 80,000 data files representing 36 human data sets and 33 experimental model data sets. The primary data sets include genomic, transcriptomic, proteomic, metabolomic, and epigenomic data of several thousand human brain samples. These are augmented by additional human molecular data sets derived from blood and/or cerebrospinal fluid, and preclinical experimental model systems including human‐derived induced pluripotent stem cells as well as mouse, rat, and *Drosophila* models.

Content in the portal is annotated with a controlled metadata vocabulary that enables indexing for search and query. Data files can be downloaded via the underlying data store, Synapse (https://synapse.org; Friend & Norman, 2013), using an established set of APIs and access tools. Synapse uses sophisticated data governance functions that enable all users to view the entire range of available data and content and request additional access to any controlled human subjects data as relevant.

In this article, we present step‐by‐step protocols that detail how to find and download AD Knowledge Portal data. Basic Protocol 1 focuses on downloading genomic variant and RNA‐seq data from the same individuals within a selected study using a web interface. Basic Protocol 2 demonstrates how to use programmatic clients to download files. Finally, Basic Protocol 3 details how to work with file annotations and metadata that describe files, samples, and individuals.

## Basic Protocol 1 FIND AND DOWNLOAD FILES ASSOCIATED WITH A SELECTED STUDY

In this example, we will demonstrate how to select and download processed genomic variant, RNA‐seq, and associated metadata files from the Mayo Clinic AD Cerebral Amyloid Angiopathy Study (MC‐CAA; (https://adknowledgeportal.synapse.org/Explore/Studies/DetailsPage?Study_Name=MC‐CAA), which was generated as part of the M^2^OVE‐AD program.

### Materials

#### Hardware

Computer with Internet connection

#### Software

Up‐to‐date web browser, such as Chrome, Firefox, or Safari

**Figure 1:**
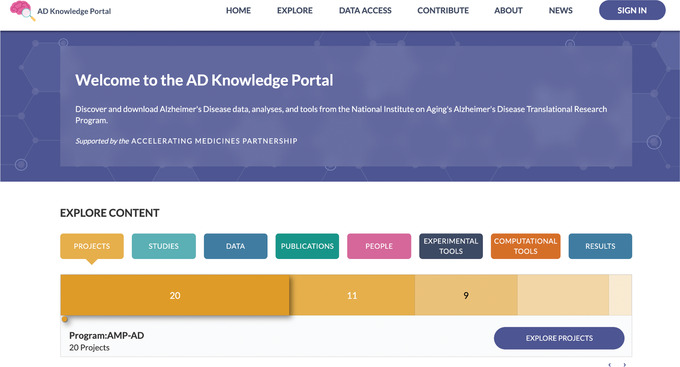
Screenshot of AD Knowledge Portal homepage, found at http://adknowledgeportal.synpase.org/.

1 Visit the AD Knowledge Portal by navigating to https://adknowledgeportal.synapse.org (Fig. 1).
  The protocol steps and screenshots in this article refer to Site Version 1.4.17.

**Figure 2:**
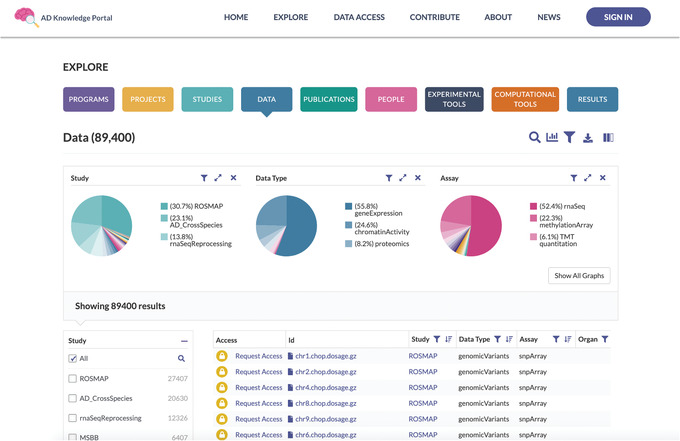
Screenshot of the Data Table, found at http://adknowledgeportal.synpase.org/Explore/Data.

2 In the navigation bar at the top, click on Explore/Data. This will take you to a new webpage, https://adknowledgeportal.synapse.org/Explore/Data, which shows a Data Table listing all files within the AD Knowledge Portal along with metadata for each file (Fig. 2).

**Figure 3:**
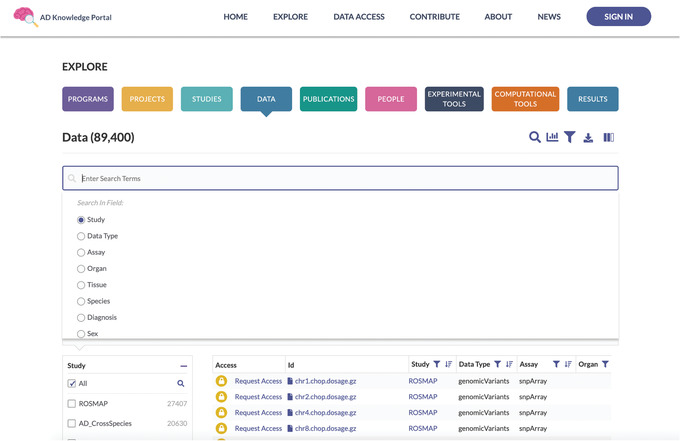
Screenshot of the Explore Data Search box, revealed by clicking on the magnifying glass icon.

**Figure 4:**
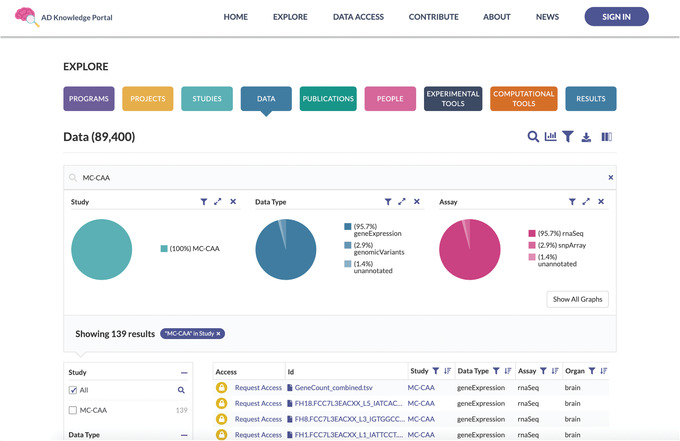
Screenshot of the Data Table after applying the “MC‐CAA” Study filter.

3 Search for files associated with a study of interest. Use the magnifying glass icon in the Explore Data Toolbar located above the pie charts to bring up the Search box (Fig. 3). Ensure that the *Study* facet is selected in the Search in Field list below the Search box. Type the value “MC‐CAA” into the Search box and hit the Return or Enter button on your keyboard. This will update the Data Table to show only files from the MC‐CAA study (Fig. 4).
  The Explore Data section presents several ways to select data files of interest. The top of the page displays pie charts that summarize the number of files based on file annotations of interest, including Study, Data Type, Assay, Organism, and Tissue, among others. Selection of one of these chart segments will filter the table below to select just that set of files. Access a full list of values for a specific annotation by clicking the funnel‐shaped Filter icon in the Chart toolbar. Alternatively, access the filters using the facet selection boxes to the left of the Data Table.

**Figure 5:**
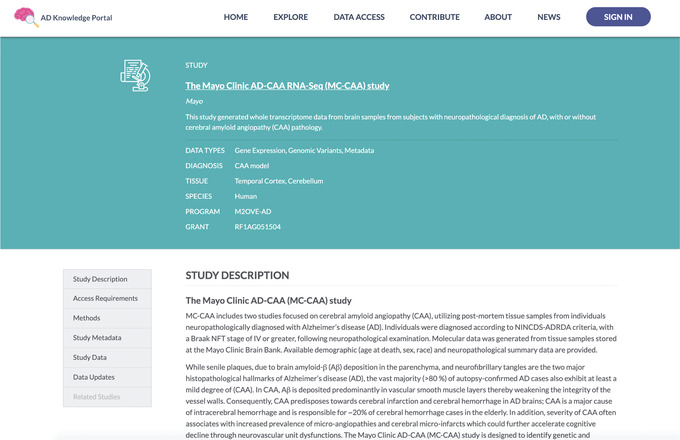
Screenshot of the MC‐CAA Study Detail page, found at http://adknowledgeportal.synapse.org/Explore/Studies/DetailsPage?Study_Name=MC‐CAA.

4 Learn more about the selected study. Within the Data Table, click the “*MC‐CAA*” link in the *Study* column. This will take you to a Study Details page (https://adknowledgeportal.synapse.org/Explore/Studies/DetailsPage?Study_Name=MC‐CAA), which presents information about the study goals, subject selection criteria, and methodological details of the biological assays used. The Study Details pages also display lists of metadata and data associated with the study (Fig. 5).

**Figure 6:**
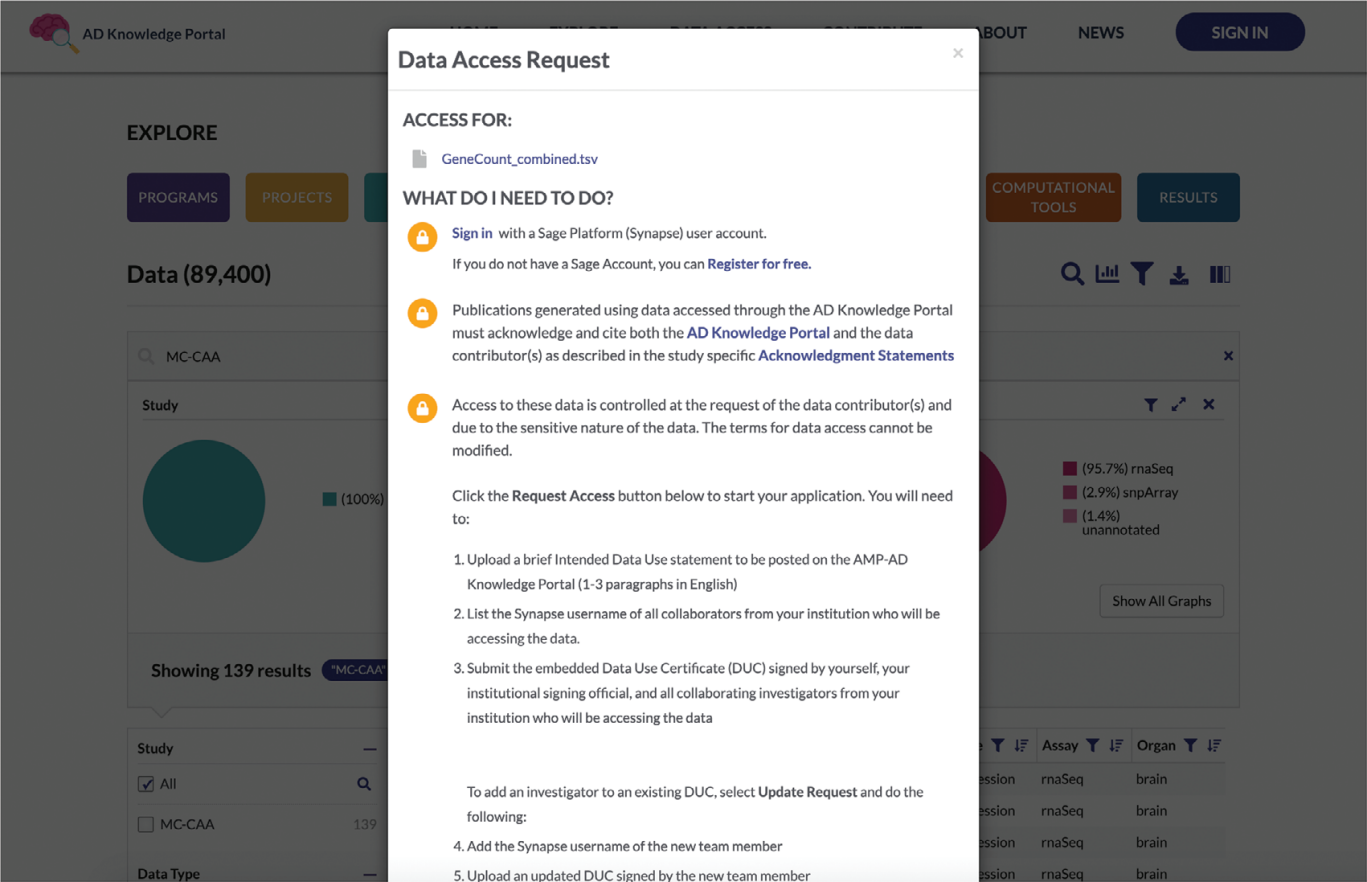
Screenshot of Access Request dialog box, revealed after clicking on Request Access in the Data Table.

5 Log in or create an account to get access to files. Returning to the Data Table, examine the Access column on the left. Logged‐in users should see View Terms next to a green open lock icon. New users will see a yellow lock icon with a Request Access message. Click on Request Access to bring up a dialog box with a list of steps needed to get access to the file of interest (Fig. 6).
  - Register for an account or log in to access data. Data in the AD Knowledge Portal are hosted in the data repository, Synapse (https://www.synapse.org). Initiate the account creation process by clicking on “Sign in with a Sage Platform (Synapse) user account.” This will take you to a registration page within Synapse. Enter your e‐mail address and click “Send registration info.”
    Synapse also supports login using a Google account. Visit the Synapse docs site to learn more: https://docs.synapse.org/articles/user_profiles.html#adding‐google‐email‐addresses‐to‐enable‐sso.
  - Check your e‐mail and follow the steps in the e‐mail sent from *noreply@synapse.org* to create your account.
  - For controlled data from human subjects, you will need to apply to get access to controlled data. Complete and submit the Data Use Certificate (DUC) following the instructions to Request Access.
  - Agree to the AD Knowledge Portal Data Use Terms by reading the statement that describes how to acknowledge data in the Portal and clicking I Accept Terms of Use.
  - Once you have access, log in to the site and proceed with the following steps.

**Figure 7:**
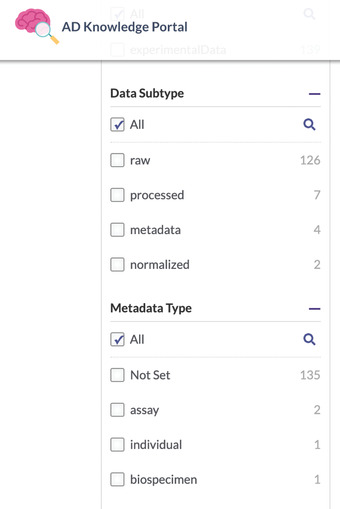
Screenshot of the *Data Subtype* and *Metadata Type* facet selection boxes, two of the facets that can be found to the left of the Data Table.

6 Use additional filters to select specific files of interest. Using the facet list on the left of the Data Table, locate the *Data Subtype* facet (Fig. 7). Check the value “*processed*.”
  Data Subtype (also referred to as dataSubtype) is a file annotation that indicates if data in the file are raw, processed, or normalized, or if the file contains metadata (see Basic Protocol 3 for more information about working with metadata). In this example, we are selecting processed RNA‐seq gene count matrices and genomic variant PLINK files. While beginning analysis with unmanipulated gene counts (as designated by the “processed” annotation) is often preferred, you may instead check the value “normalized” to find the RNA‐seq gene counts normalized by conditional quantile normalization (CQN).

**Figure 8:**
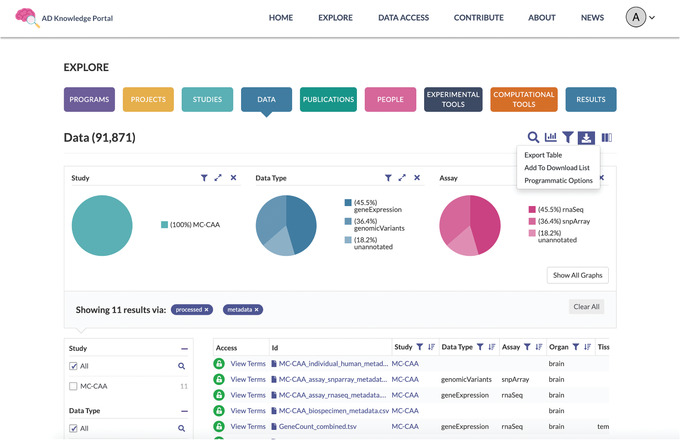
Screenshot of the download menu found within the Explore Data Page toolbar. Three possible options are: Export Table, Add to Download List, or Programmatic Options.

7 Select the metadata files associated with the MC‐CAA study. Using the facet list on the left of the Data Table, locate the *Data Subtype* facet (Fig. 7). Check the value “*metadata*” to add these to the Data Table. The Data Table will now contain both “processed” and “metadata” files.
8 Add selected files from the Data Table to the Download List. Using the Explore Data Page toolbar above the Pie Charts, click on the Download Icon (Fig. 8).
  - From the drop‐down menu, select Add to Download List. This will bring up a new notification bar that reports the number and size of files you have selected.
  - Select the **Add** button on the right of the notification bar to add these files to the Download List.

**Figure 9:**
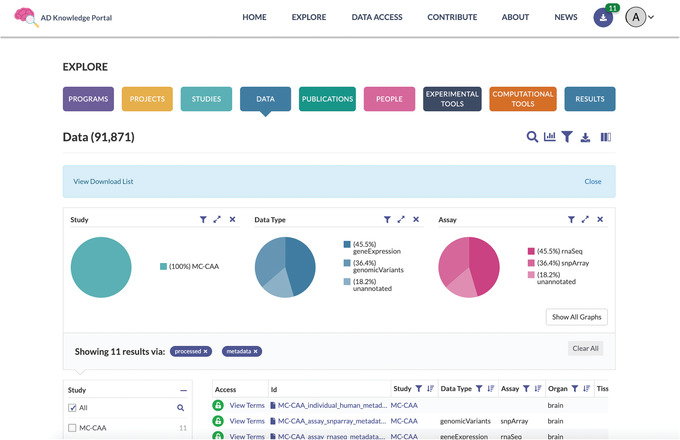
Screenshot of Explore Data Page after adding files to the Download List; two ways to access the Download List are depicted.

9 Download files. Access the download list by selecting View Download List from the notification bar, or by clicking the Download List icon at the top of the site (Fig. 9).
  - Enter a name for your package in the PackageFileName text box.
  - Click the Create Package button on the bottom right of the Download List.
  - After the package has been created, the notification bar will be updated. Click Download Package to download a zip file to your computer.
    Before downloading any data onto your computer, ensure that your work environment is secure and that you understand how to manage data that may contain private health information (PHI). Note that there is a limitation on the number and size of files that can be downloaded through the web; see Basic Protocol 2 for programmatic download options.

10 Download file annotations, which are required for interpreting the data files. The utility of file annotations is explained in more detail in Basic Protocol 3. Using the menu bar above the Pie Charts, click on the Download Icon (Fig. 8).
  - From the drop‐down menu, select Export Table. This will bring up a new dialog box. Customize the file type and contents using the selections and checkboxes.
  - Click Next to generate the file. This will bring up a new dialog box that indicates the file is ready for download.
  - Click Download to download the file to your computer. The resulting file contains a list of file identifiers along with file annotations.

## Basic Protocol 2 DOWNLOAD FILES IN BULK USING THE COMMAND LINE CLIENT

An alternative to downloading files through the web is the use of programmatic clients that directly access files in the Portal's underlying data store, Synapse. Programmatic clients include command line, R, and Python. Using programmatic clients is more efficient for downloading a large number of files or downloading files of a large size. Using programmatic clients also promotes reproducibility by recording which data were used for analysis. The Synapse command line client synapseclient can be used to download all data and file annotations from the portal with a single command. This protocol can be used instead of steps 6‐9 in Basic Protocol 1.

An important consideration when determining which Synapse programmatic client to use to download data is download speed. The command line and Python synapseclient support multithreaded download, and will provide faster download speeds than the Synapse R client. Here, we detail how to use the command line client. To use Python or R clients, follow the steps outlined in the Synapse documentation: https://docs.synapse.org/articles/tutorial‐download‐data‐portal.html

### Materials

#### Hardware

Computer with Internet connection and sufficient privileges to install additional software

#### Software

Up‐to‐date web browser, such as Chrome, Firefox, or Safari
Python 3 (https://www.python.org/downloads/)
synapseclient package version 2.2 or higher

1 Install Python 3 (https://www.python.org/downloads/). The command line synapseclient is installed with the Synapse Python client; therefore, Python 3 is required to install the synapseclient package.
2 Install the synapseclient package by following the steps outlined in the Synapse documentation: http://python‐docs.synapse.org/build/html/index.html#installation.
3 Login to Synapse by following the steps outlined in the documentation: https://python‐docs.synapse.org/build/html/CommandLineClient.html#login. If working on your personal computer, you may store your credentials locally by including the ‐‐rememberMe argument to allow automatic authentication with future Synapse interactions. This is recommended to prevent a case where you might accidentally share your password while sharing analytical code. In almost all cases, your Synapse API key is more secure than your password, and is recommended to be used to login. Find your API key in your user Profile: https://docs.synapse.org/articles/user_profiles.html#api‐key.
synapse login ‐u <Synapse username> ‐p <API key> ‐‐rememberMe
4 From Explore Data in the portal, select the Download Options icon and Programmatic Options to visualize the command to download the data subset (see Fig. 8).
5 The command synapse get with the ‐q argument downloads files from the entirety of the portal data that meet the specified condition. See the synapse get documentation for more detail: https://python‐docs.synapse.org/build/html/CommandLineClient.html#get. In this example, all “*processed*” and “*metadata*” files from the “*MC‐CAA*” study are downloaded. Execute the following command from the directory where you would like to store the files.
synapse get ‐q "SELECT * FROM syn11346063 WHERE (("study" = 'MC‐CAA') AND ("dataSubtype" = 'processed' OR "dataSubtype" = 'metadata'))"
6 Also, in your working directory, you will find a SYNAPSE_TABLE_QUERY_###.csv file that lists the annotations associated with each downloaded file. This is the same information that can be obtained through the web interface using Export Table (Basic Protocol 1, step 10). Here, you will find helpful experimental details relevant to how the data were processed. Additionally, you will find important details about the file itself including the file version number.

## Basic Protocol 3 WORKING WITH FILE ANNOTATIONS AND METADATA

File metadata in the form of annotations provide critical information about the data in the AD Knowledge portal. Annotations describe content within the files, including type of data and assay, tissue of origin, source study, and ID of the source individual. Metadata conform to a structured schema to ensure that the data are searchable and standardized across studies and programs. The standards for defining, managing, and implementing controlled vocabularies for content in Synapse are available in the synapseAnnotations Github repository (https://github.com/Sage‐Bionetworks/synapseAnnotations).

While a subset of metadata is stored as annotations on the file, the complete metadata are stored within a set of comma‐separated values (CSV) files. The complete metadata expand on the annotation set with extra details about the individuals, specimens (or biosamples) from the individuals, and the assay performed on the specimens. There are typically several metadata CSV files required to fully represent the details of a single individual, specimen, or assay. These can be combined with the file annotations to get the complete details about the file. The individual metadata file describes each individual in the study. Each row corresponds to a unique individual identifiable by the key *individualID*. The biospecimen metadata file describes the specimens that were collected, and includes metadata such as tissue type, cell type, or nucleic acid source. While each row corresponds to a unique specimen identified by the key *specimenID*, there may be multiple specimens per individual. The biospecimen file also contains the key *individualID*; thus, the individual metadata file can be joined on this key. The assay metadata file contains experimental processing details such as technology used, batch processing, quality control metrics, and the key *specimenID*. If specimens or individuals were characterized across several assay types, there may be more than one assay metadata file.

In short, metadata files of the individual, biospecimen, and assay type can be joined together on the keys *individualID* and *specimenID*. This protocol demonstrates how to use R software to join multiple files.

### Materials

#### Hardware

Computer with Internet connection

#### Software

Up‐to‐date web browser, such as Chrome, Firefox, or Safari
R software (R Core Team, 2020)
tidyverse package for R (Wickham et al., 2019)

1 Ensure that you have the metadata files using steps 7‐10 in Basic Protocol 1 or step 5 in Basic Protocol 2.
2 Install and load the tidyverse package in R to perform data frame manipulations.
install.packages("tidyverse")
library(tidyverse)
3 While reading in each metadata file with read_csv, specify the column types as character with “c”. Consistent column types ensure that common variables can be joined. This code joins data frames using all variables in common across the individual, biospecimen, and assay metadata files. A right_join preserves only the individuals and biospecimens that are characterized in each assay type: RNA‐seq and SNP array.
individual <‐ read_csv("MC‐CAA_individual_human_metadata.csv",
  col_types = cols(.default = "c")
)
biospecimen <‐ read_csv("MC‐CAA_biospecimen_metadata.csv",
  col_types = cols(.default = "c")
)
rnaseq_assay <‐ read_csv("MC‐CAA_assay_RNAseq_metadata.csv",
  col_types = cols(.default = "c")
)
snparray_assay <‐ read_csv("MC‐CAA_assay_snpArray_metadata.csv",
  col_types = cols(.default = "c")
)
RNASeq <‐ reduce(
  list(individual, biospecimen, rnaseq_assay),
  right_join
)
snpArray <‐ reduce(
  list(individual, biospecimen, snparray_assay),
  right_join
)

## COMMENTARY

### Troubleshooting

There are several resources for obtaining additional help and support while using the AD Knowledge Portal. There is an FAQ page on the website that outlines common questions and answers. In addition, the AD Knowledge Portal has a well‐used discussion forum (https://www.synapse.org/#!Synapse:syn2580853/discussion/default) where users can post questions as well as view previous discussion threads. For questions about the underlying Synapse data store and working with Synapse programmatic clients, we recommend visiting the Synapse docs site: https://docs.synapse.org/. Finally, for questions that aren't answered elsewhere, users can contact the AD Knowledge Portal team directly via the “Contact Us” link on the website.

### Author Contributions

**Anna K. Greenwood**: Conceptualization; project administration; writing‐original draft; writing‐review & editing. **Kelsey S. Montgomery**: Formal analysis; methodology; resources; validation; writing‐original draft. **Nicole Kauer**: Data curation; methodology; resources; validation; writing‐review & editing. **Kara H. Woo**: Data curation; methodology; resources; validation; writing‐review & editing. **Zoe J. Leanza**: Project administration; validation; writing‐review & editing. **William L. Poehlman**: Methodology; writing‐review & editing. **Jake Gockley**: Methodology; writing‐review & editing. **Solveig K. Sieberts**: Conceptualization; methodology; resources; writing‐review & editing. **Ljubomir Bradic**: Conceptualization; formal analysis; project administration; visualization; writing‐review & editing. **Benjamin A. Logsdon**: Conceptualization; formal analysis; methodology; project administration; resources; validation; writing‐review & editing. **Mette A. Peters**: Conceptualization; data curation; methodology; project administration; resources; validation; writing‐review & editing. **Larsson Omberg**: Conceptualization; methodology; project administration; resources; supervision; validation; writing‐review & editing. **Lara M. Mangravite**: Conceptualization; funding acquisition; project administration; writing‐original draft; writing‐review & editing
